# Decoding the Malignant Potential of Hydatidiform Moles through Fibroblast and LAIR2 Signatures

**DOI:** 10.34133/cancomm.0038

**Published:** 2026-07-07

**Authors:** Chen Li, Zhiang Chen, Jiandong Chen, Na Yu, Shuai Zhang, Weiguo Lu, Songfa Zhang, Jiale Qin

**Affiliations:** ^1^ Department of Human Genetics, and Women’s Hospital, Zhejiang University School of Medicine and Zhejiang Provincial Key Laboratory of Genetic and Developmental Disorders, Hangzhou, Zhejiang, P. R. China.; ^2^ Department of Ultrasound, Women’s Hospital, Zhejiang University School of Medicine, Hangzhou, Zhejiang, P. R. China.; ^3^ Forensic Science Center of Zhejiang University, Hangzhou, Zhejiang, P. R. China.; ^4^ Department of Gynecologic Oncology, Women’s Hospital, Zhejiang University School of Medicine, Hangzhou, Zhejiang, P. R. China.; ^5^ Zhejiang Provincial Clinical Research Center for Gynecological Disease, Hangzhou, Zhejiang, P. R. China.; ^6^ Zhejiang Key Laboratory of Maternal and Infant Health, Hangzhou, Zhejiang, P. R. China.; ^7^ Zhejiang Provincial Key Laboratory of Precision Diagnosis and Therapy for Major Gynecological Diseases, Hangzhou, Zhejiang, P. R. China.

Hydatidiform mole (HM) is a benign form of gestational trophoblastic disease (GTD) caused by abnormal fertilization. Although initially noninvasive, HM carries a substantial risk of malignant progression. Following uterine evacuation, approximately 13% to 20% of complete HMs (CHMs) and 0.5% to 5% of partial HMs (PHMs) progress to post-molar gestational trophoblastic neoplasia (pGTN) [1,2]. Notably, CHM carries a substantially higher risk of malignant progression than do non-molar pregnancy events, including miscarriage, full-term pregnancy, or termination of pregnancy.

The mechanisms underlying the progression of CHM to pGTN remain poorly understood. Advanced maternal age (>40 years), pre-evacuation serum human chorionic gonadotropin (hCG) levels >100,000 IU/l, uterine enlargement, and large theca lutein cysts were previously considered clinical risk factors for local invasion and/or distant metastasis [1], but consensus is not reached. With the widespread use of high-resolution ultrasound and sensitive hCG assays, CHM is frequently diagnosed at earlier gestational stages, leading to a reduced prevalence of these high-risk clinical features. Nevertheless, the incidence of pGTN has not shown a corresponding decrease [3]. This discrepancy indicates that malignant progression cannot be fully attributed to the clinical presentation at diagnosis and may instead be indicative of the inherent biological characteristics of the mole [4,5].

To investigate this, we prospectively collected chorionic villous tissue at the time of uterine evacuation from 10 CHM patients, including 4 cases that subsequently developed pGTN (pGTN group) and 6 cases that experienced spontaneous regression (SR group). All samples underwent single-cell transcriptomic profiling (Fig. 1A, Table S1, and Supplementary Materials and Methods). After quality control, excluding cells with >25% mitochondrial transcripts, fewer than 500 detected genes, and genes expressed in fewer than 3 cells, a total of 43,402 cells from the SR group and 48,778 from the pGTN group were retained. Following batch-effect correction and unsupervised clustering, 7 distinct cell populations were identified. These comprised 3 trophoblast subtypes, cytotrophoblast (CTB), syncytiotrophoblast (STB), and extravillous trophoblast (EVT), and 4 non-trophoblast populations: fibroblast (FB), endothelial cell (Endo), Hofbauer cell (HC), and immune cell (Immune) (Fig. 1B). Cell-type annotation was based on established canonical markers, including paternally expressed 10 (*PEG10*) for CTBs, chorionic gonadotropin α subunit (*CGA*) for STBs, and major histocompatibility complex, class I, G (*HLA-G*) for EVTs (Table S2) [6].

**Fig. 1.**
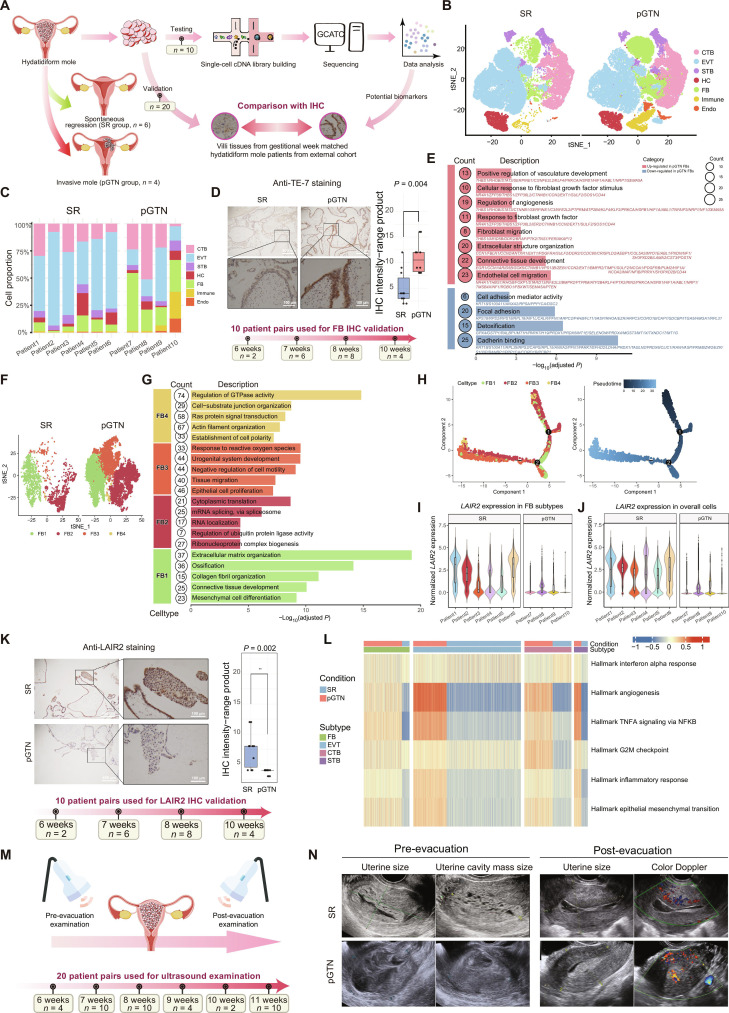
Characterization of villous tissues from complete hydatidiform mole (CHM) patients with subsequent spontaneous regression (SR) and post-molar gestational trophoblastic neoplasia (pGTN) outcomes. (A) Study design. Schematic representation of the prospective collection and processing of chorionic villous tissues for single-cell transcriptomic profiling and immunohistochemistry (IHC). The workflow illustrates the comprehensive process from sample collection to data analysis for both SR and pGTN groups. (B) Single-cell transcriptomic profiling of total cells. Unsupervised clustering followed by t-distributed stochastic neighbor embedding (t-SNE) dimensionality reduction were performed on all retained cells. Seven distinct cell populations were identified, including 3 trophoblast subtypes and 4 nontrophoblast populations. Colors indicate distinct cell clusters. (C) Cell proportion distribution across individual samples. Cell-type proportions were calculated from single-cell transcriptomic profiling data and displayed as stacked bar plots. Seven annotated cell populations showed variable distributions among samples, with marked differences in cellular composition between SR and pGTN groups. Colors denote the cell types defined in (B). (D) Validation of fibroblast abundance. IHC staining for the fibroblast-specific antigen TE-7 was performed on formalin-fixed, paraffin-embedded (FFPE) sections from an independent validation cohort (10 patient pairs). pGTN cases demonstrated significantly greater staining area and stronger intensity than did the SR group, validating the increased fibroblast abundance (*P* = 0.004). (E) Functional enrichment of pGTN fibroblasts. Gene Ontology (GO) functional enrichment analysis was performed on significantly differentially expressed genes (DEGs) in pGTN fibroblasts. The analysis revealed enhanced angiogenic activity and extracellular matrix (ECM) synthesis, together with down-regulated cell adhesion pathways in the pGTN group. (F) Subclustering of fibroblasts. Fibroblasts were subjected to unsupervised reclustering followed by t-SNE projection. Four distinct subtypes (FB1 to FB4) were identified, with different subtype distributions observed between SR and pGTN samples. Colors indicate the 4 fibroblast subtypes. (G) Functional characterization of fibroblast subtypes. GO enrichment analysis was applied to significantly up-regulated genes specific to each fibroblast subtype. The plot illustrates the functional differences among fibroblast subtypes, with distinct biological functions identified for each subtype. Colors correspond to the subtypes defined in (F). (H) Developmental trajectory of fibroblast subtypes. Pseudotime trajectory analysis of fibroblast subtypes was conducted using Monocle 2. The trajectory positions FB1, FB2, and FB4 at the root, progressing toward the terminal FB3 state. The trajectory line represents the inferred developmental progression. (I) *LAIR2* expression in fibroblasts. Comparative gene expression analysis of single-cell transcriptomic data between the SR and pGTN groups within the fibroblast population was performed. The violin plots demonstrate the comparative expression levels of *LAIR2* in fibroblasts extracted from each sample, highlighting its down-regulation in the pGTN group. (J) *LAIR2* expression in all cells. Comparative gene expression analysis of single-cell transcriptomic data between the SR and pGTN groups across all cells. The violin plots show *LAIR2* expression levels, demonstrating consistent down-regulation in the pGTN group compared to the SR group. (K) Validation of LAIR2 protein expression. Semiquantitative IHC staining for LAIR2 was performed on FFPE sections from the matched validation cohort (10 patient pairs). Consistent with transcriptomic findings, LAIR2 protein expression was markedly reduced in the pGTN group (*P* = 0.002). (L) Pathway activation in trophoblasts and fibroblasts. Pseudo-bulk gene set variation analysis (GSVA) was conducted on 3 trophoblast subtypes and fibroblasts. The analysis revealed consistent up-regulation of gene sets related to angiogenesis and epithelial–mesenchymal transition in the pGTN group. Colors indicate the GSVA enrichment scores, ranging from low (blue) to high (red). (M) Ultrasound imaging study design. Transvaginal color Doppler ultrasonography was performed 5 to 7 d post-evacuation in an independent, gestational age-matched cohort of 20 CHM patient pairs (±2 d) who progressed to pGTN or achieved SR. The schematic shows the ultrasound examination timeline for patients at different gestational ages. (N) Ultrasonographic features of myometrial vascularity. Evaluation of myometrial hypervascular focus presence via transvaginal color Doppler ultrasonography. Myometrial hypervascular focus was more frequently detected in the pGTN group, serving as a relative marker of early vascular remodeling. Images display representative color Doppler signal clusters that indicate hypervascularity. CTB, cytotrophoblast; EVT, extravillous trophoblast; STB, syncytiotrophoblast; HC, Hofbauer cell; FB, fibroblast; Immune, immune cell; Endo, endothelial cell.

Notably, FBs were significantly more abundant in all 4 cases of the pGTN group compared with the SR group, as determined by a bootstrap-derived confidence interval permutation test (Fig. 1C). The findings were further validated by immunohistochemical (IHC) staining for the FB-specific antigen TE-7 in an independent cohort of 10 gestational age-matched pairs of post-evacuation (±2 d) and formalin-fixed paraffin-embedded villous tissues from CHM patients (1 pair at 6 weeks, 3 pairs at 7 weeks, 4 pairs at 8 weeks, and 2 pairs at 10 weeks). Using a semiquantitative scoring system independently assessed by 2 blinded pathologists (*κ* = 0.845; Fig. S1, Table S3, and Supplementary Materials and Methods) [7], pGTN cases demonstrated significantly broader staining areas and stronger intensity (*P* < 0.05), confirming increased FB abundance independent of gestational age (Fig. 1D).

Differentially expressed gene analysis demonstrated increased angiogenic activity and a stronger propensity for extracellular matrix (ECM) synthesis and remodeling in pGTN FBs. Concurrent down-regulation of cell adhesion pathways indicated a shift toward a pro-invasive phenotype, in which reduced cellular attachment facilitates active myometrial infiltration and ECM reorganization (Fig. 1E). Reclustering analysis identified 4 distinct FB subtypes (FB1 to FB4) (Fig. 1F, Table S4, and Supplementary Materials and Methods). Quantitative assessment of stromal composition in pGTN revealed marked pathogenic remodeling: Although canonical matrix-producing FB1 and biosynthetic FB2 subtypes remained highly abundant, the malignant microenvironment was uniquely characterized by a striking expansion of the FB3 subtype. Functional enrichment analysis indicated that FB1 was associated with ECM production and connective tissue development, FB2 with active RNA metabolism and protein translation, FB3 with oxidative stress responses and tissue migration, and FB4 with tissue remodeling (Fig. 1G). Pseudotime analysis positioned FB1, FB2, and FB4 at the root of the differentiation trajectory, progressing toward the terminal FB3 state (Fig. 1H). These findings suggest that the pGTN microenvironment actively promotes this transition, driving expansion of the migratory, stress-responsive FB3 subtype and restructuring stromal architecture to facilitate invasion.

Comparative gene expression analysis between 2 groups identified leukocyte-associated immunoglobulin-like receptor 2 (*LAIR2*) as significantly and consistently down-regulated in the pGTN group, with a log_2_ fold change (log_2_FC) > 0.5 and a Bonferroni-adjusted *P* < 0.05 (Fig. 1I and J and Table S5). Correlation analysis further confirmed that the reduced *LAIR2* signature and increased FB abundance were independent of patient age and differentiation status (Fig. S2). LAIR2 is a trophoblast-derived molecule specifically expressed by EVT subtypes (Figs. S3 and S4), which is involved in placental angiogenesis during implantation, local immune regulation, and spiral artery remodeling in early pregnancy [8,9]. Its down-regulation in the pGTN group at evacuation suggests impaired EVT function during a critical window of vascular transformation, potentially leading to inadequate placental invasion and a microenvironment conducive to malignant progression in CHM.

To evaluate the clinical relevance of LAIR2 in subsequent pGTN development, semiquantitative IHC staining was performed on the same 10 gestational age-matched villous tissue pairs previously assessed for TE-7 (Table S3). Consistent with transcriptomic findings, LAIR2 protein expression was markedly reduced in the pGTN group, reflected by significantly lower combined intensity and extent scores (independently evaluated by pathologists, *κ* = 0.926; anti-LAIR2; Fig. 1K). Pseudo-bulk gene set variation analysis further revealed consistent up-regulation of gene sets related to angiogenesis and epithelial–mesenchymal transition across multiple trophoblastic cell types and FBs in the pGTN group, implicating enhanced angiogenesis and ECM remodeling in malignant progression (Fig. 1L).

To further characterize angiogenesis-related features in the pGTN group, we analyzed transvaginal color Doppler ultrasonography performed 5 to 7 d post-evacuation in an independent, gestational age-matched cohort of 20 CHM patient pairs (±2 d) who progressed to pGTN or achieved SR. The cohort comprised 2 pairs at 6 weeks, 5 pairs at 7 weeks, 5 pairs at 8 weeks, 2 pairs at 9 weeks, 1 pair at 10 weeks, and 5 pairs at 11 weeks of gestation (Fig. 1M). The presence of myometrial hypervascular focus was evaluated (Table S6) and was detected more frequently in the pGTN group than in those who achieved SR. This imaging feature may serve as a relative marker of early trophoblast-associated vascular remodeling during CHM peri-evacuation period (Fig. 1N).

In summary, systematic analyses of placental villi at evacuation revealed distinct FB infiltration patterns and differential LAIR2 expression between CHM patients who underwent SR and those who experienced malignant progression. These differences were consistent at both the transcriptomic and protein levels and were accompanied by alterations in stromal and trophoblastic composition. Moreover, the presence of post-evacuation myometrial vascular invasion was markedly associated with subsequent pGTN, underscoring a link between early stromal remodeling and malignant progression. We postulate that the intrinsic loss of the maternally expressed cell-cycle inhibitor *CDKN1C* (*p57*) in androgenetic CHM likely drives the aberrant co-expansion of FB1 and FB2 subtypes. Coupled with microenvironmental oxidative stress, this remodeling promotes the accumulation of the stress-responsive FB3, creating a niche that impairs trophoblast differentiation and facilitates invasion. Together, these results implicate remodeling and angiogenesis as key pathogenic features of malignant progression. Further functional investigations and large-scale validation are required to confirm the robustness and clinical utility of these signatures.

## Ethical Approval

This study was approved by the Ethics Committee of Women’s Hospital, Zhejiang University School of Medicine (reference: IRB-20210167-R and 20180128) and was performed in accordance with the principles of the Helsinki Declaration. The participants provided informed consent prior to beginning the study.

## Data Availability

The raw single-cell transcriptomic data have been uploaded to National Genomics Data Center (NGDC) of China under accession number HRA014340. Any additional information required to reanalyze the data reported in this paper is available from the lead contact upon reasonable request.
