# Supplementary material for: Decoding the Malignant Potential of Hydatidiform Moles through Fibroblast and LAIR2 Signatures
Source: Cancer Commun (Lond). 2026 Jul 7;46:0038. doi: 10.34133/cancomm.0038 (PMC13338560; doi:10.34133/cancomm.0038)
Supplement: Supplementary 1 — Materials and Methods Figs. S1 to S4 Tables S1 to S6 [file cancomm.0038.f1.zip › 00089-Supplementary Materials-final.docx]

**Supplementary Material for**

**Decoding the malignant potential of hydatidiform moles through fibroblast and LAIR2 signatures**

Chen Li^1,†^, Zhiang Chen^1,†^, Jiandong Chen^1^, Na Yu^2^, Shuai Zhang^3^, Weiguo Lu^4,5,6^, Songfa Zhang^4,*^, Jiale Qin^2,5,7,*^

1. Department of Human Genetics, and Women’s Hospital, Zhejiang University School of Medicine & Zhejiang Provincial Key Laboratory of Genetic and Developmental Disorders, Hangzhou, Zhejiang, P. R. China.
2. Department of Ultrasound, Women’s Hospital, Zhejiang University School of Medicine, Hangzhou, Zhejiang, P. R. China.
3. Forensic Science Center of Zhejiang University, Hangzhou, Zhejiang, P. R. China.
4. Department of Gynecologic Oncology, Women’s Hospital, Zhejiang University School of Medicine, Hangzhou, Zhejiang, P. R. China.
5. Zhejiang Provincial Clinical Research Center for Gynecological Disease, Hangzhou, Zhejiang, P. R. China.
6. Zhejiang Key Laboratory of Maternal and Infant Health, Hangzhou, Zhejiang, P. R. China.
7. Zhejiang Provincial Key Laboratory of Precision Diagnosis and Therapy for Major Gynecological Diseases, Hangzhou, Zhejiang, P. R. China.

^†^These authors contributed equally to this work.

**^*^Corresponding authors:**

J.L.Q. (Jiale Qin), Email: [qinjiale@zju.edu.cn](mailto:qinjiale@zju.edu.cn).

S.F.Z. (Songfa Zhang), Email: [zhangsongfa@zju.edu.cn](mailto:zhangsongfa@zju.edu.cn).

## Supplementary Materials and Methods

### Single-cell transcriptomic data processing and annotation

To ensure the reproducibility of cell type definitions, we identified cluster-specific marker genes using a Wilcoxon rank-sum test with Bonferroni correction. A finding was considered credible only if it met the following pre-specified thresholds: adjusted *P* < 0.05, average log2 fold-change (log2FC) > 0.5, and a minimum percentage (min.pct) of cells expressing the gene > 0.25. The complete list of marker genes for all major cell types is provided in Supplementary Table S2. Cell type annotations were assigned based on canonical markers, including platelet and endothelial cell adhesion molecule 1 (*PECAM1*; Endothelial); protein tyrosine phosphatase receptor type C (*PTPRC*; Immune); Thy-1 cell surface antigen (*THY1*), collagen type I alpha 1 chain (*COL1A1*), collagen type I alpha 2 chain (*COL1A2*), and actin alpha 2, smooth muscle (*ACTA2*; Fibroblasts); CD14 molecule (*CD14*), allograft inflammatory factor 1 (*AIF1*), CD163 molecule (*CD163*; Hofbauer cells); chorionic gonadotropin alpha subunit (*CGA*), chorionic gonadotropin subunit beta 3 (*CGB3*; STBs); major histocompatibility complex, class I, G (*HLA-G*), integrin subunit alpha 5 (*ITGA5*; EVTs); PAGE family member 4 (*PAGE4*), paternally expressed 10 (*PEG10*; CTBs); and keratin 7 (*KRT7*; Trophoblasts). Finally, cluster robustness was verified by examining sample contribution to rule out patient-specific bias, and further validated by integrating additional samples, which confirmed that the core cell identity and compositional patterns remained stable.

### Identification of fibroblast subtypes and signatures

For fibroblast subtyping, cells annotated as fibroblasts were subsetted and subjected to unsupervised reclustering using a resolution of 0.1, which yielded four distinct clusters (FB1–FB4). To characterize their biological functions, we identified differentially expressed genes (DEGs) for each subtype using the FindAllMarkers function with the thresholds: adjusted *P* < 0.05, log2FC > 0.5, and min.pct > 0.25. These DEGs were then subjected to functional enrichment analysis using Gene Ontology (GO) and Kyoto Encyclopedia of Genes and Genomes (KEGG) to define the specific biological identity of each fibroblast subtype. The complete list of these defining signatures is included in Supplementary Table S4.

### Trajectory analysis

Trajectory analysis was performed using Monocle 2 (v2.24.0). We selected high-dispersion genes for ordering based on the criteria: mean_expression ≥ 0.1 and dispersion_empirical ≥ 1× dispersion_fit. Dimensionality reduction was conducted using the DDRTree algorithm with max_components = 2. State 1 was defined as the root of the trajectory, as this state was predominantly enriched with FB2 cells exhibiting progenitor-like biological functions.

### Functional enrichment analysis

Functional enrichment analyses were performed using a Hypergeometric test, with significance defined by a Benjamini-Hochberg (BH) corrected false discovery rate (FDR) < 0.05. For Gene Set Variation Analysis (GSVA), we utilized gene sets from MSigDB v2024.1 (subcollections: c2.cp.kegg, c5.go, c6, and h). GSVA scores were calculated using a Gaussian kernel, and differential pathway activities between groups were assessed using the Limma package (Empirical Bayes t-test), with a significance threshold of BH-adjusted FDR < 0.05.

### Cell proportion analysis

The proportions of cell subtypes between spontaneous regression (SR) and post-molar gestational trophoblastic neoplasia (pGTN) samples were compared using permutation testing implemented in the scProportionTest R package (v0.0.0.9000, https://github.com/rpolicastro/scProportionTest). Differences in cell abundance were evaluated using a permutation test (*n* = 1,000 shuffles) to calculate FDR-adjusted P-values, and a bootstrap procedure (*n* = 1,000 resamples) was implemented to generate 95% confidence intervals.

### Ultrasound imaging analysis

To better delineate the angiogenesis-related features in the pGTN group, we employed a gestational age–matched (±2 days) and paired case-control design (pGTN:SR = 1:1) to account for physiological changes in uterine vascularity with gestation.

Ultrasound data were obtained from the GTD imaging prospective cohort at the Women’s Hospital, Zhejiang University School of Medicine (ethics approval No. 20180128). Patients were included in this study according to the following criteria:

1. Clearly defined clinical outcome according to International Federation of Gynecology and Obstetrics (FIGO) 2021 clinical guidelines [1];
2. Standardized ultrasound timing, with transvaginal color Doppler ultrasonography performed within 3 days before evacuation and repeated at 5-7 days after evacuation, and complete image data available;
3. Accurate gestational age determination from a clearly reported last menstrual period and regular cycles.

The presence of myometrial hypervascular focus was assessed and defined as an abnormal course and clustering of arcuate arteries within the inner one-third of the myometrium adjacent to the endometrium, characterized by tortuous, disorganized vessels forming confluent Doppler signal clusters. This feature may serve as a relative marker of early trophoblastic-related vascular remodeling during the peri-evacuation period of molar pregnancy.

### Immunohistochemical staining and analysis

To validate fibroblasts and LAIR2 expression in real-world clinical settings, we employed a gestational age–matched (±2 days) and outcome-matched (pGTN:SR = 1:1) comparison strategy. This approach was adopted to account for gestational age-related physiological changes in uterine vascularity and tissue composition. The formalin-fixed and paraffin-embedded (FFPE) tissue samples were obtained from the biobank of the Women’s Hospital, Zhejiang University School of Medicine. FFPE sections (4 μm thickness) were deparaffinised in xylene, rehydrated in a graded ethanol series, and quenched with endogenous peroxides. The sections were then incubated with fibroblast-specific antigen TE‑7 (1:50, ab207178, Abcam) and anti-LAIR2 (1:600, TA350703, OriGene). Phosphate-buffered saline was used as the negative control.

We have explicitly defined the semi-quantitative scoring criteria "Intensity-Range Product", yielding a total score from 0 to 16. The intensity was classified into five grades: 0, negative; 1, weak; 2, moderate; 3, strong; and 4, very strong. The proportion of positively stained cells was also divided into five classes: 0, ≤5%; 1, 6%-25%; 2, 26%-50%; 3, 51%-75%; and 4, >75%. The IHC staining scores were obtained by multiplying the intensity score with the proportion score (Supplementary Figure S1). All the staining results were evaluated independently by two experienced pathologists blinded to the clinical data [2]. Inter-observer agreement was assessed using weighted Cohen’s kappa statistics.

## Supplementary Figures


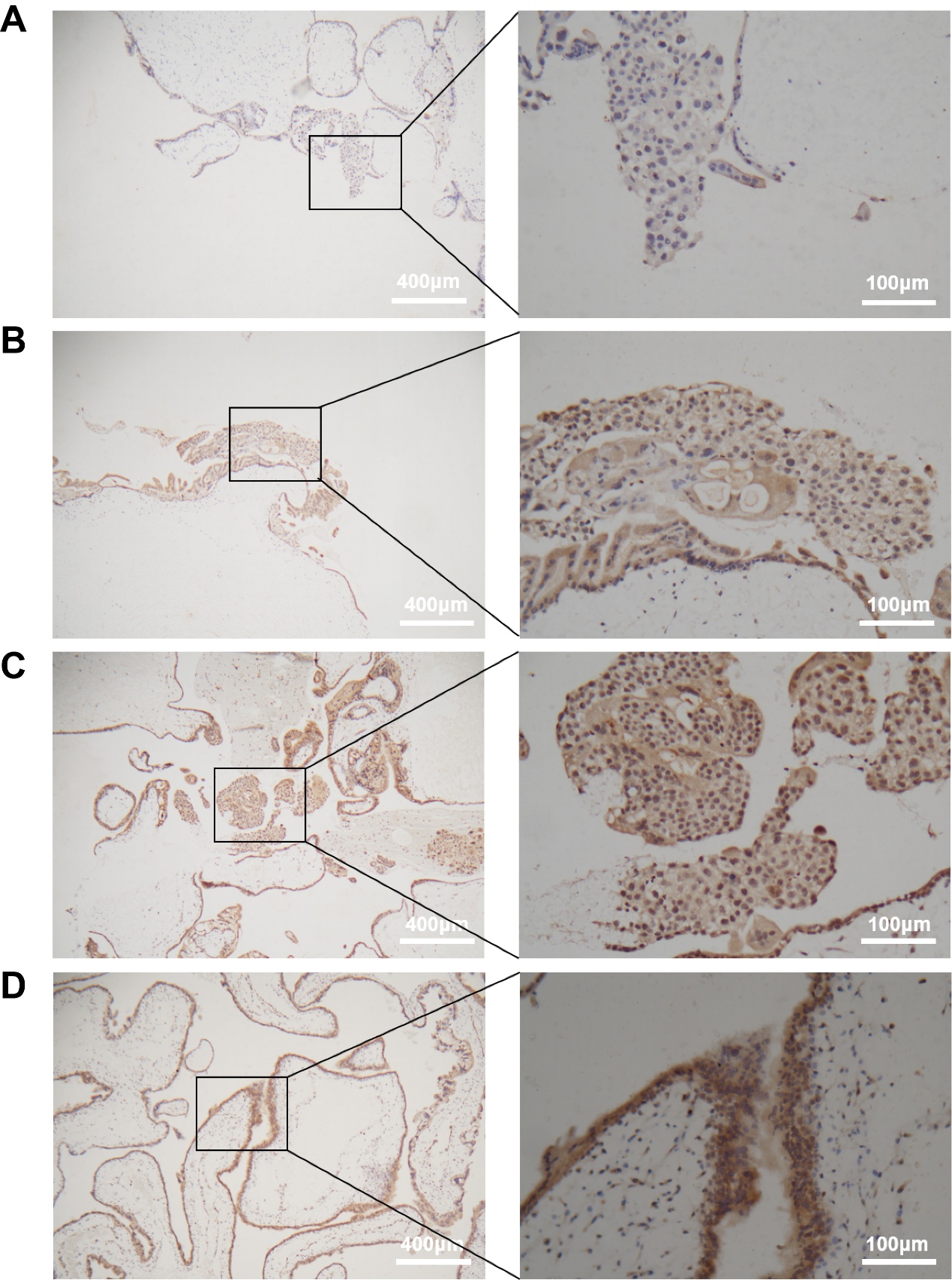


**Supplementary Figure S1. Representative micrographs and scoring thresholds for immunohistochemical analysis.**

IHC staining of FFPE sections was semi-quantitatively evaluated by two blinded pathologists to calculate the final immunoreactivity score. The figure demonstrates the graded criteria, where the immunoreactivity score was calculated by multiplying the staining intensity grade by the percentage of positive cells grade.

1. Score 4: Weak intensity (grade 1) in >75% of cells (grade 4). Calculation: 1 × 4 = 4.
2. Score 8: Moderate intensity (grade 2) in >75% of cells (grade 4). Calculation: 2 × 4 = 8.
3. Score 12: Strong intensity (grade 3) in >75% of cells (grade 4). Calculation: 3 × 4 = 12.
4. Score 16: Very strong intensity (grade 4) in >75% of cells (grade 4). Calculation: 4 × 4 = 16.

Abbreviations: IHC, Immunohistochemistry; FFPE, formalin-fixed, paraffin-embedded.

**
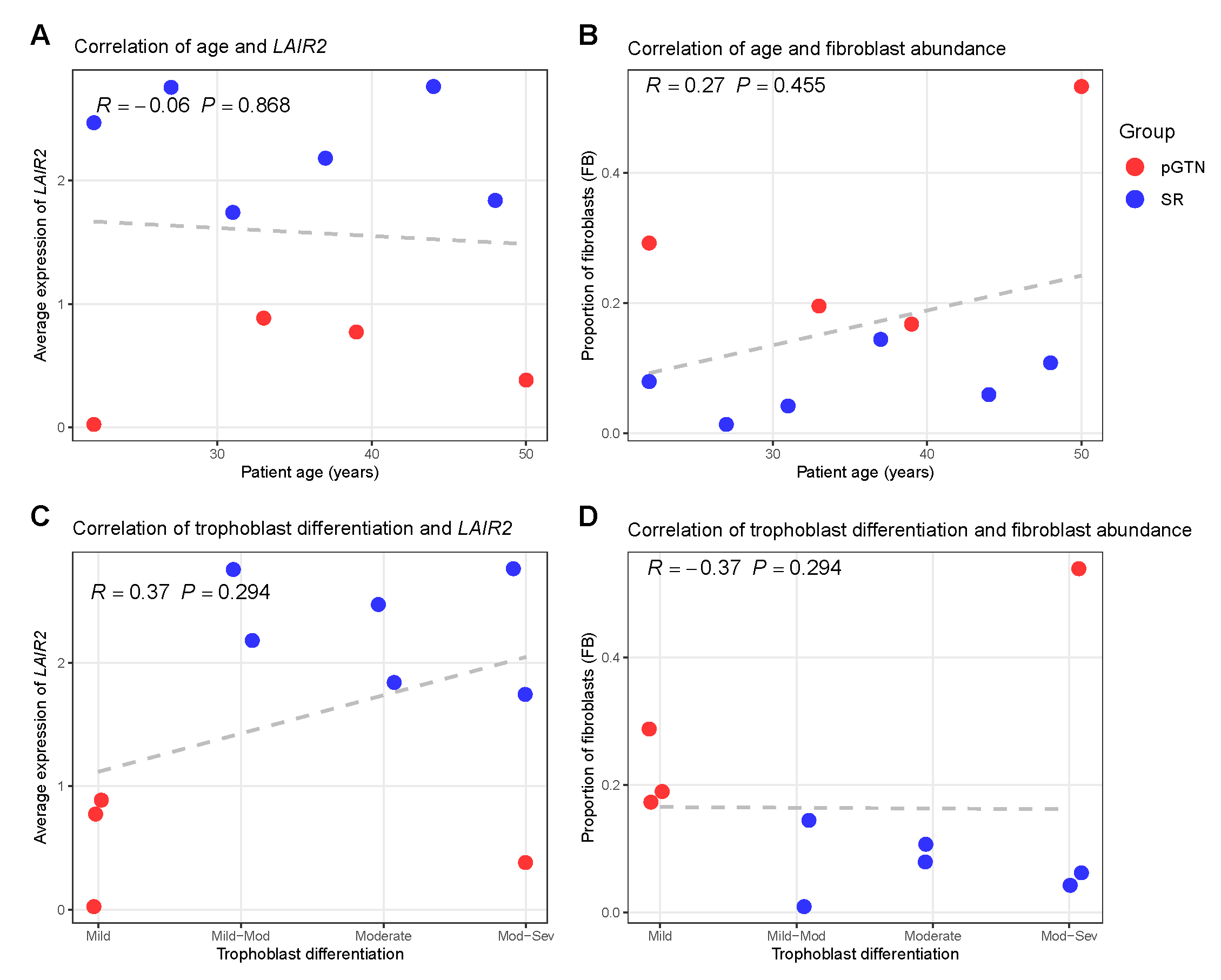
**

**Supplementary Figure S2. Correlation of patient age and trophoblast differentiation with molecular features.**

Spearman’s rank correlation analysis was applied to data from the single-cell discovery cohort (*n* = 10). The lack of significant correlations indicates that the identified molecular signatures (reduced *LAIR2* expression and increased FB abundance) are independent of both patient age and differentiation status.

1. Correlation of patient age and LAIR2 expression.
2. Correlation of patient age and fibroblast abundance.
3. Correlation of trophoblast differentiation and LAIR2 expression.
4. Correlation of trophoblast differentiation and fibroblast abundance.

The X-axes represent patient age (A, B) or trophoblast differentiation (C, D), and the Y-axes represent LAIR2 expression (A, C) or fibroblast abundance (B, D). R represents Spearman’s rank correlation coefficient. *P* > 0.05 indicates no statistical significance. Points are colored by prognosis (Red: pGTN; Blue: SR).

Abbreviations: SR, spontaneous regression; pGTN, post-molar gestational trophoblastic neoplasia; FB, fibroblast; *LAIR2*, leukocyte associated immunoglobulin like receptor 2.


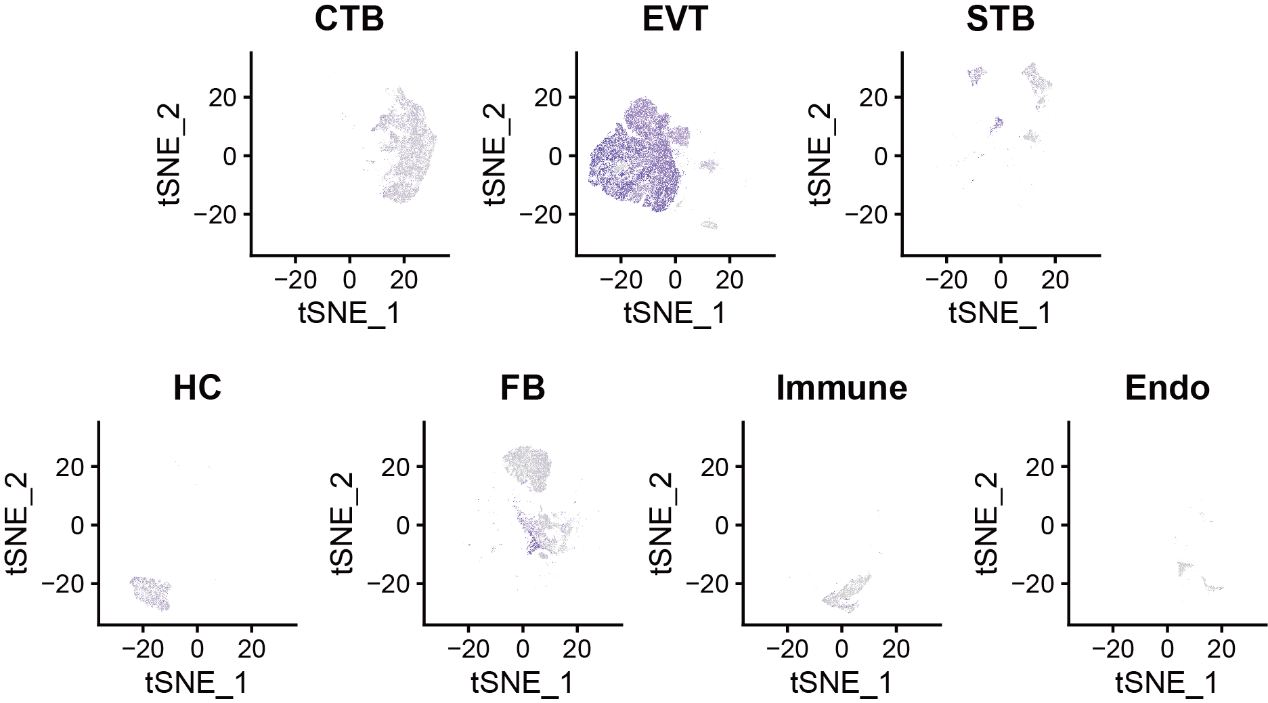


**Supplementary Figure S3. Specificity of *LAIR2* expression across major cell types.**

Feature plot of *LAIR2* expression visualized on t-SNE projections across all identified major cell types. The expression pattern demonstrates that *LAIR2* is predominantly enriched in the EVT population, confirming its specific role as an EVT-specific marker rather than a general trophoblast molecule in this dataset. Purple intensity represents the normalized expression level of *LAIR2*.

Abbreviations: *LAIR2*, leukocyte associated immunoglobulin like receptor 2; t-SNE, t-distributed stochastic neighbor embedding; CTB, cytotrophoblast; EVT, extravillous trophoblast; STB, syncytiotrophoblast; HC, Hofbauer cell; FB, fibroblast; Immune, immune cell; Endo, endothelial cell.


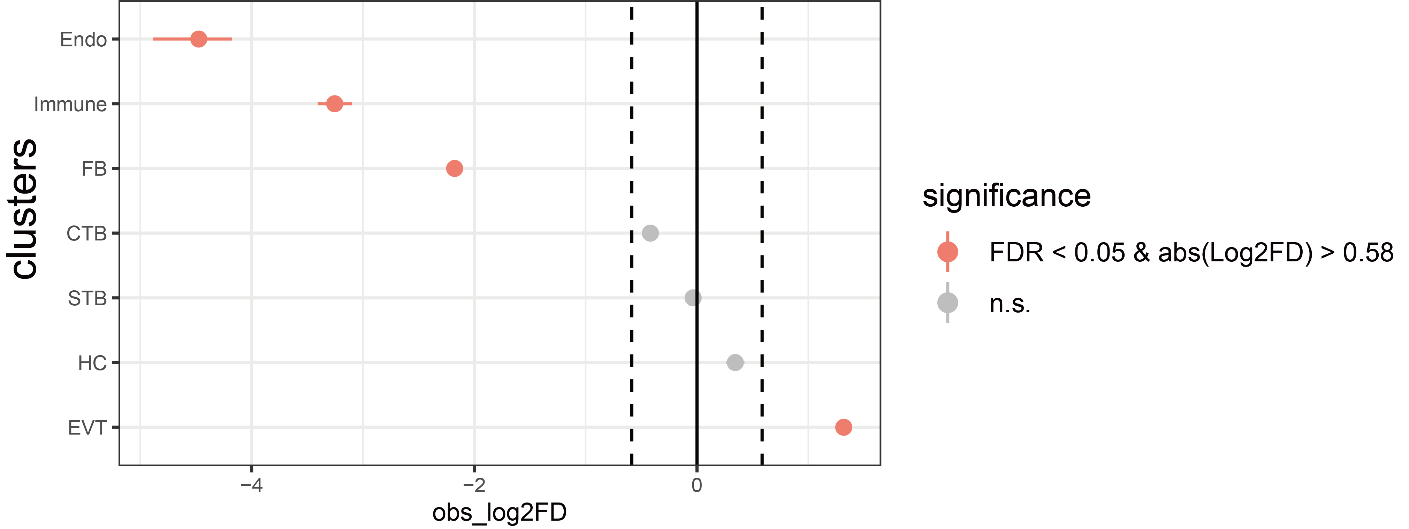


**Supplementary Figure S4. Differential abundance of major cell types between SR and pGTN groups.**

Differential abundance analysis of cell proportions was conducted using a bootstrap-derived confidence interval permutation test (*n* = 10 samples). The forest plot shows significantly altered cell populations between the two groups, indicating compositional changes in the cellular microenvironment. The X-axis represents the Log2FD (SR vs pGTN). Dots represent the Log2FD, and error bars indicate the 95% confidence intervals. Negative values (left) indicate cell types enriched in the pGTN group, whereas positive values (right) indicate those enriched in the SR group.

Abbreviations: SR, spontaneous regression; pGTN, post-molar gestational trophoblastic neoplasia; CTB, cytotrophoblast; EVT, extravillous trophoblast; STB, syncytiotrophoblast; HC, Hofbauer cell; FB, fibroblast; Immune, immune cell; Endo, endothelial cell; FDR, false discovery rate; Log2FD, Log2 fold difference; n.s., not significant.

**Supplementary Table S1. Clinical information of patients included in single-cell transcriptomic profiling.**

[Excel]

**Supplementary Table S2.** **Canonical marker genes utilized for major cell type annotation.**

[Excel]

**Supplementary Table S3. Characteristics of the 10 patient pairs used for FB and LAIR2 IHC validation.**

[Excel]

**Supplementary Table S4.** **Marker genes defining the four distinct fibroblast subtypes and statistics of differential gene expression.**

[Excel]

**Supplementary Table S5. Results of DEG analyses both within FB subtype and across the entire cell types**

[Excel]

**Supplementary Table S6.** **Patient information and myometrial blood flow findings from ultrasound examinations.**

[Excel]

**References:**

1. Ngan HYS, Seckl MJ, Berkowitz RS, Xiang Y, Golfier F, Sekharan PK, et al. Update on the diagnosis and management of gestational trophoblastic disease. Int J Gynaecol Obstet. 2018;143:79-85.

2. Li C, Chen J, Wu H, Zhang S, Yu N, Chen Z, et al. Dissecting trophoblastic heterogeneity in abnormal pregnancy: Insights from comparative analysis of twin-pregnancy with hydatidiform mole and coexisting live fetus. Genes Dis. 2026;13(1):101651.
